# Knowledge, Attitude, and Practice of Thai Dairy Farmers on the Use of Antibiotics

**DOI:** 10.1155/2024/5553760

**Published:** 2024-06-28

**Authors:** Niorn Ratanapob, Aksorn Saengtienchai, Theera Rukkwamsuk

**Affiliations:** ^1^ Department of Large Animal and Wildlife Clinical Sciences Faculty of Veterinary Medicine Kasetsart University, Kamphaeng Saen Campus, 1 Malaiman Road, Kamphaeng Saen, Nakhon Pathom 73140, Thailand; ^2^ Department of Pharmacology Faculty of Veterinary Medicine Kasetsart University, 50 Ngamwongwan Road, Chatuchak, Bangkok 19000, Thailand

## Abstract

Antibiotics have been used regularly in dairy farms by veterinarians; however, they were also used occasionally by farmers without any veterinary prescriptions. Because knowledge, attitude, and practice are important for sustainable antibiotic use, the levels of these aspects among farmers and associated factors should be determined to improve antibiotic use in dairy farming. The study was carried out in 89 Thai dairy farmers, using a structured questionnaire. Data were analyzed using descriptive statistics. The questions were scored and the total scores were calculated for each aspect. Factors associated with knowledge, attitude, and practice scores were identified using the multivariate linear regression. The mean scores for knowledge, attitude, and practice were 62%, 86%, and 78%, respectively. Higher education, participation in a training related to antibiotic use, and being supervised by the Veterinary Teaching Hospital Nong Pho were associated with a higher knowledge score (*p* < 0.050). Farmers with less experience received a higher attitude score (*p* = 0.020). Acquiring antibiotic knowledge from other farmers was associated with a lower practice score (*p* = 0.005). A positive association was found between knowledge and attitude scores (*p* = 0.021) and practice and attitude score (*p* < 0.001). Due to the insufficient knowledge on antibiotic use, there is a need to fill the gap to ensure prudent antibiotic use by farmers. Recommendations including training farmers by veterinarian to perceive the prudent antibiotic uses, encouraging young generation with higher education to participate in dairy farming, providing antibiotic use protocols, and decreasing the availability of antibiotics should be implemented to limit overuse by farmers.

## 1. Introduction

Antibiotics have been used frequently in humans and animals. In Thailand, antibiotics have been accessible for use in food-producing animals, such as cattle, pigs, and chickens [1]. Despite the fact that antibiotic use in dairy farms has been controlled by the Department of Livestock Development since 2003, almost all dairy farmers experienced a poor use of antibiotics [2]. In dairy cattle, antibiotics are used primarily for therapeutic purposes; however, they are sometimes used for prophylaxis purposes, such as to prevent postpartum metritis and mastitis [2, 3]. A previous study in Northern Thailand found that blanket use of antibiotics for dry-cow therapy was performed by almost one-third of farmers [2]. Nonjudicious use of antibiotics induces development of antimicrobial resistance, which is now considered a global health concern. Livestock farmers in most developing countries have been considered to be significant imprudent antibiotic users [4]. The decrease in antibiotic use in food animals has been considered a major measure to preserve the efficacy of antibiotics [5]. However, based on a survey in Northern Thailand, a considerable proportion of farmers were unaware of antimicrobial resistance [2].

Antimicrobial resistance has been reported in Thai dairy cattle [6–8], especially in farms in which antibiotics have been extremely used [6]. On the basis of the experience of local dairy veterinarians and laboratory results, antimicrobial resistance has also existed in the western part of the country. Some pathogens that carry resistance genes are environmental bacteria [6], which can be easily exposed by farmers. Antimicrobial resistance genes were also detected in raw milk and could be transferred to consumers if products are not properly processed [9]. The COVID-19 pandemic reminds us that incurable infectious diseases may be caused by the evolution of existing pathogens, including antimicrobial resistant microorganisms [10]. Therefore, antimicrobial resistance in dairy cattle must be explored actively to guarantee health security. In addition, the Thai government has launched the National Stretegic Plan on Antimicrobial Resistance 2023–2027. The plan consists of 6 strategies, including antimicrobial resistance surveillance using One Health approach; regulation of antimicrobial distribution; infection prevention and control, and antimicrobial stewardship; antimicrobial containment, and antimicrobial use in agriculture and animals; public awareness on antimicrobial resistance and prudent use of antimicrobials; and governance mechanisms to implement and sustain antimicrobial actions.

A previous study in human health revealed that the knowledge and attitude about antibiotic use of physicians was associated with their prescribing behavior [11]. The prescribing behavior of veterinarians was also associated with participating in a training on antibiotic use and antimicrobial resistance [12]. According to studies in dairy farms, the behaviors of antibiotic use of farmers were also related to their knowledge and attitude [13, 14]. The knowledge, attitude, and practice on antibiotic use of farmers have been considered determinants of the development of antibiotic resistance [15]. Therefore, correcting farmers' knowledge, attitude, and practice are required for sustainable antibiotic use. Identification of factors related to the knowledge, attitude, and practice on antibiotic use of dairy farmers is still limited. Age, gender, and level of education of farmers were found to be associated with knowledge, attitudes, and practices of dairy farmers [16]. However, all of them are endogenous factors which cannot be modified to improve knowledge, attitudes, and practices. For this reason, more research is meaningful to determine factors which can be adjusted and consequently promote knowledge, attitudes, and practices.

Examining knowledge, attitude, and practice related to antibiotic use of dairy farmers will be a fundamental approach to promote appropriateness of antibiotic use. Benefits will be expanded by identifying factors associated with these aspects, especially if the factors are modifiable. The flaws revealed from this procedure will lead to appropriate interventions for farmers to become prudent antibiotic users [4, 14, 16, 17]. The objective of this study was to determine the knowledge, attitude, and practice of dairy farmers and their associated factors on the use of antibiotics.

## 2. Materials and Methods

### 2.1. Data Collection

A structured questionnaire was designed to evaluate knowledge, attitude, and practice related to antibiotic use in dairy farmers in Thai language. It was pretested by three expertized ruminant veterinarians and four experienced dairy farmers. A minor revision was conducted to clarify some points and provide more possible answer options. The final version questionnaire consisted of 4 sections: (1) general information about the farm and the farmer (6 questions); (2) knowledge (8 questions); (3) attitude (10 questions); and (4) practice (17 questions). In total, there were 11 open-ended questions and 32 closed-ended questions. Some of them were multiple-true-false questions, especially in the knowledge section.

**Figure 1:**
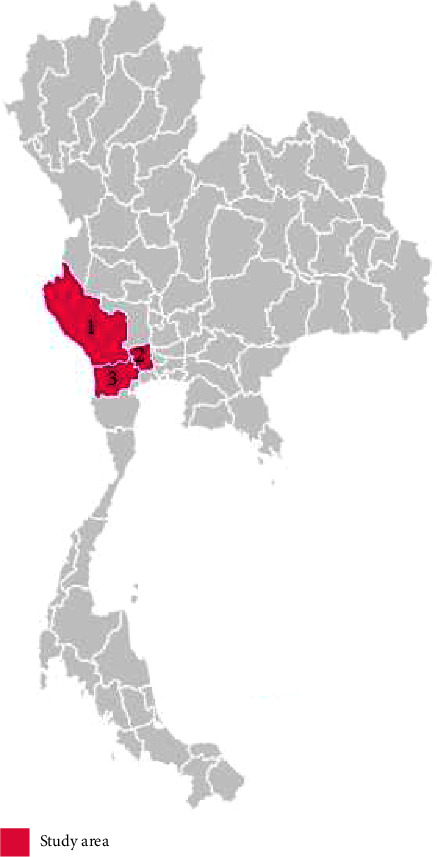
Study area including Kanchanaburi Province (1), Nakhon Pathom Province (2), and Ratchaburi Province (3).

The target population included all 109 farms for which health management had been provided by Kasetsart University Veterinary Teaching Hospital (KUVTH) at Kamphaeng Saen, Nakhon Pathom Province (27 farms), and at Nong Pho, Ratchaburi Province (82 farms). The study included Nakhon Pathom, Ratchaburi, and Kanchanaburi provinces (Figure 1). The principal investigator and eight well-trained veterinarians completed the questionnaire by face-to-face interview during October 2020 to June 2021. The study protocol was approved by the Kasetsart University Research Ethics Committee (KUREC-SS64/218), and it was conducted in accordance with the approved protocol.

### 2.2. Data Analyses

Data were entered into Microsoft Excel (Microsoft Inc., Redmond, WA) before importing to Stata 14 (StataCorp LP, College Station, TX, US). Descriptive analyses were performed to determine percentages and means (standard deviation; SD) for categorical and continuous variables, respectively. The answers of each question were scored as 0 if it represents poor knowledge, attitude, or practice, 0.5 if it represents intermediate knowledge, attitude, or practice, and 1 if it represents good knowledge, attitude, or practice. However, six questions in the practice section could not be scored because they are not judgeable, such as the availability of other drug groups on the farm and the most commonly used antibiotics. The total score for each farmer was then calculated separately for each section. It was calculated only for respondents who answered all the questions in that section. The most likely total score of the knowledge, attitude, and practice sections was 0 to 32, 0 to 10, and 0 to 11, respectively.

Before further analyses were performed, continuous variables in the general information section were categorized, and some categorical variables were recategorized. Multivariate linear ordinary least squares regression was conducted to identify factors associated with total knowledge, attitude, and practice scores separately. All variables in the general information section, including gender, age, level of education, experience in dairy farming of the respondent, herd size, and sources of antibiotic knowledge, were variables of interest, except variables having less than 10% variation. Other variables of interest included the hospital that cares for the farm and the total score of other sections. Manual backward elimination was used for model building. Variables with *p* ≥ 0.05 were removed from the model until the *p* value of all remaining variables was less than 0.05. Possible two-way interactions and confounders were evaluated for each final model. The final models were diagnosed to ensure that the model assumptions were not violated. Multiple comparisons were made for categorical variables with more than two classes in the final models using the Bonferroni adjustment.

## 3. Results

### 3.1. General Information

Of 109 eligible dairy farmers, 89 (81.65%) could participate in the present study. The response rates of farms under supervision of KUVTH Kamphaeng Saen and Nong Pho were 74.07% (20/27) and 84.14% (69/82), respectively. Due to the COVID-19 lockdown, 20 farmers could not be reached. Almost two-thirds of the respondents were male. The mean age of the respondents was 48 (SD = 10.4) years, ranging from 25 to 70 years. Most of the respondents (71.92%) did not have bachelor's degrees. The mean experience in dairy cattle farming was 18 (SD = 8.9) years, ranging from 2 to 42 years. The minimum and maximum herd sizes were 12 and 160 cows, respectively, with a mean of 51 (SD = 32). The most common sources of knowledge on antibiotic use were from veterinarians of KUVTH, other farmers, and drug stores (Table 1).

### 3.2. Knowledge

**Figure 2:**
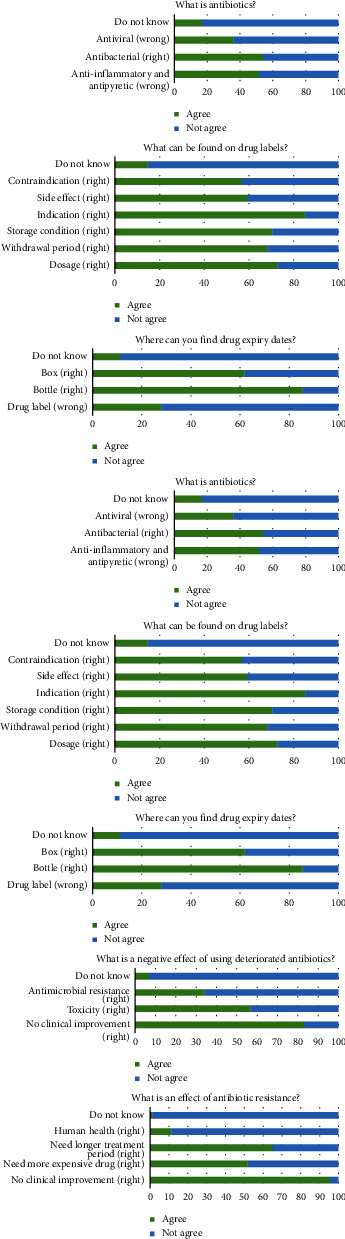
Frequency distribution for 8 questions in the knowledge section from 89 dairy farmers.

Only 16.85% of the respondents had the correct information that antibiotics have only bacterial killing activity. Some respondents (14.61%) did not know what information was included on the drug label. Most respondents (88.76%) knew where they could find an antibiotic expiration date. High temperature and sunlight were recognized by more than 80% of respondents as factors affecting antibiotic stability, whereas physical contamination and improper humidity were not considered by most respondents. The majority of the respondents recognized that a refrigerator provides appropriate conditions for antibiotics (88.76%), while 40.45% believed that antibiotics could be kept in the environmental condition of Thailand. Most of the respondents (96.63%) knew how to notice deteriorated antibiotics. According to knowledge related to antimicrobial resistance, 65.91% did not know that the use of degraded antibiotics could lead to antimicrobial resistance, and only 11.24% realized the effects of antimicrobial resistance in animals on human health (Figure 2).

The total knowledge scores ranged between 4 and 30 out of 32, with a mean of 19.80 (SD = 6.39) or 61.88%. The total score was significantly associated with farmer education (*p* = 0.026), antibiotic knowledge acquisition from the training course (*p* = 0.001), the hospital caring for the farm (*p* = 0.016), and the total attitude score (*p* = 0.021) (Table 2). Based on multiple comparisons, farmers with lower or upper high school degrees and bachelor or higher degree gained a significantly higher total knowledge score compared to farmers with elementary education (grade 6) or lower (*p* = 031 and 0.013, respectively). Participating in a training course related to antibiotic use is also associated with a higher knowledge score. Farmers under KUVTH Nong Pho supervision received a higher total score in this section than farmers under KUVTH Kamphaeng Saen supervision. In addition, farmers who gained a higher total knowledge score also obtained a higher total attitude score.

### 3.3. Attitude

**Figure 3:**
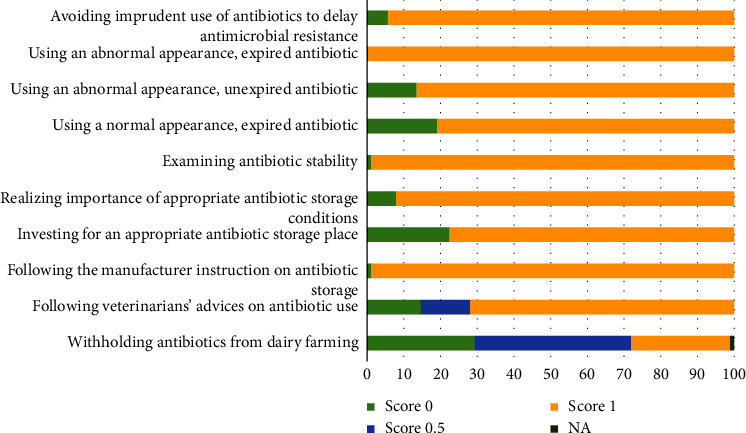
Frequency distribution of scores for questions in the attitude section from 89 dairy farmers.

More than 70% of farmers thought that withholding antibiotic treatment from their farms would negatively affect animal health and productivities. Most farmers respected veterinarians' advice on antibiotic use (71.91%), and 98.88% paid attention to appropriate antibiotic storage following the manufacturer instruction. Although most farmers realized the importance of examining the expiration date and/or physical appearance of antibiotics stored long before use (98.88%), 19.10% thought that expired antibiotics could be used if their appearances are still normal. If they were advised by a veterinarian, only a few respondents (5.62%) did not recognize the importance of avoiding irrational antibiotic use to postpone the development of antimicrobial resistance (Figure 3).

The frequency distribution of the scores for each question in the attitude section is presented in Figure 3. The total attitude scores ranged from 6 to 10. The mean total score in this section was 8.57 (SD = 0.86) or 85.70%. Two factors were associated with the score (Table 3), including the year of dairy farming experience (*p* = 0.020) and the total practice score (*p* < 0.001). Farmers who had a longer experience in farming (≥20 years) received a lower total attitude score compared to farmers who had a shorter experience. The total score in the attitude and practice sections was positively correlated.

### 3.4. Practice

**Figure 4:**
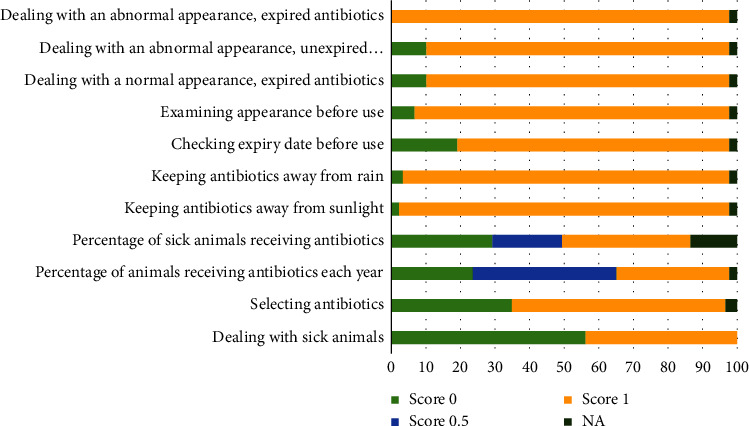
Frequency distribution of scores for questions in the practice section from 89 dairy farmers. Six questions could not be scored because they are not judgeable.

The practice section revealed that 56.18% of the respondents regularly treated their sick cattle without consulting a veterinarian, and 96.63% bought antibiotics by themselves (Figure 4). Antibiotics were chosen based mainly on the advice of veterinarians (63.95%) or farmer experience (25.58%). In addition to antibiotics (92.13%), anti-inflammatory drugs (96.63%), vitamins and minerals (95.51%), and anthelmintic drugs (88.76%) were commonly available in dairy farms. The antibiotics used the most frequently were enrofloxacin (65.17%), penicillin-streptomycin (49.44%), gentamicin (33.71%), and oxytetracycline (30.34%). The mean occurrence of disease that was treated with antibiotics estimated by farmers was 53.38% (SD = 33.66; ranged from 10% to 100%). The estimated incidence of antibiotic use per year ranged from 3.75% to 228.57% of the cattle on the farm, with a mean of 47.07% (SD = 40.82).

Two-thirds of farmers stored their leftover antibiotics in a refrigerator. However, more than half of the farmers kept the leftover antibiotics in a barn or a house. Most of the antibiotics leftover (96.55%) were not exposed directly to sunlight. However, they might be kept in a high relative humidity area and/or physically contaminated area based on the appearance of the packages. The antibiotics were kept for less than 120 days by 90.24% of the farmers. Most farmers examined an expiration date (80.46%) and observed the physical appearance of antibiotics (93.46%) every time before use and did not use antibiotics that expired (88.51%) or physically changed appearance (89.66%). No one used expired antibiotics with an abnormal appearance.

Figure 4 shows the frequency distribution of the scores for the questions in the practice section. The total practice scores ranged between 4 and 11, with a mean of 8.62 (SD = 1.20) or 78.36%. Factors associated with the total practice score were receiving knowledge about antibiotics from other dairy farmers (*p*=0.005) and the total attitude score (*p* < 0.001). Farmers who obtained knowledge from other farmers gained a lower total practice score compared to farmers who did not obtain knowledge from other farmers. The direct association was found between attitude and total practice scores (Table 4).

## 4. Discussion

### 4.1. General Information

In general, the farmers who participated in the present study were middle-aged adults (40–60 years) (62%), with low to intermediate education (72%). They had a quite long experience in dairy farming (18 years) and possessed a medium-scale farm (51 cows). Veterinarians and other farmers were found to be the most common sources of antibiotic knowledge, which was in the same trend as in the previous study [13]. The training course was another important source of antibiotic knowledge in our respondents. To provide effective training in antibiotic use, farmers need to specify topics [13]. More than a quarter of our respondents cited the Internet as a source of antibiotic information, which is visible to be higher than that found in a previous survey in Thailand [18]. This finding indicated a promising potential for the Internet as an antibiotic information provider in the future; however, the accuracy of the information must be verified.

### 4.2. Knowledge

Increasing public knowledge about antibiotics is one of the main goals of the Thailand National Strategic Plan on Antimicrobial Resistance 2023-2027. The mean total knowledge score of 62% indicated moderate knowledge about antibiotic use in our respondents. This level of knowledge may be inadequate for rational antibiotic use. Therefore, conveying precise information on antibiotics to farmers is necessary, especially about disease conditions which have to be treated with antibiotics, appropriate storage conditions, and antimicrobial resistance, which were identified as serious knowledge gaps.

A large proportion of our respondents (83%) were unable to obtain the three correct answers to the question on what antibiotics are (Figure 2). More than half (52%) thought that they have anti-inflammatory and antipyretic properties. The misconception of anti-inflammatory properties was found in 61% of Turkish farmers [15], but in a Malaysian study, it was less than 50% [19]. Almost all farmers in the previous study knew that antibiotics do not have antiviral properties [14], whereas in the present study, 36% of our respondents believed that antibiotics could kill viruses. Almost half (54%) of the respondents in the present study knew that antibiotics work to combat bacteria, which is obviously lower than the 90% reported in previous reports [4, 14]. Better antibiotic knowledge was found in urban residents compared to rural residents [15]. The proportion of respondents who knew that antibiotics have no anti-inflammatory and antiviral properties reported by a comprehensive survey in Thailand was lower than those found in the present study (43% vs. 48% and 50% vs. 64%), although some respondents of the previous study were urban residents [18]. Fifteen percent of our respondents did not know what information is on the drug labels. They may not pay attention to the text because it contains some technical terms that are difficult to understand [13]. Old-aged and illiterate farmers might also be responsible for this neglect. Therefore, improving drug labels should be concerned.

Most farmers were unaware that humidity and physical contamination can degrade antibiotics. These misunderstandings also need to be corrected. Almost 90% of the farmers in the northern region of Thailand knew that antimicrobial resistance could be induced by antibiotic use in animals [2]. However, the majority of the respondents in the present study (66%) did not know that the use of deteriorated antibiotics could lead to the development of antimicrobial resistance in cattle. The proportion of farmers concerned that antimicrobial resistance in animals could pose a threat to human health ranged from 5% to 61% [2, 14–16, 19–21]. In the present study, 11% of the respondents knew about this issue. These findings indicate an urgent need to educate dairy farmers on antimicrobial resistance to delay expansion.

### 4.3. Attitude

On average, the total attitude score was at an acceptable level (86%) with a low variation. Nevertheless, adjusting all misbeliefs would be of interest. Almost all farmers who participated in a study in Northern Thailand thought that antibiotics were important for livestock farming [2]. In the present study, 29% of farmers believed that without antibiotics, their farms could be discontinued. For Swedish dairy farmers, it was uncertain to pursue agriculture without antibiotics [14]. A considerable proportion of our respondents (43%) though indicated that animal performance would be negatively affected by antibiotic restriction on the farm. This finding is relevant to previous studies in which farmers were concerned that restriction of antibiotic use would produce more detrimental than beneficial effects, including on animal welfare [15, 16, 22]. However, if proper management practices are implemented in combination with reducing antibiotic use, negative effects on animal performance can be controlled [17]. Appropriate nutritional, housing, health, and biosecurity management, genetic selection, alternative therapy, and prompt disease identification have been considered as possible approaches to reduce antibiotic use [13, 15, 16, 22]. It is important to notify this information and provide support to farmers to decrease their dependence on antibiotics.

Veterinarians' advice was perceived by our respondents as trustworthy information on antibiotic use, which is consistent with previous reports [13, 23]. Therefore, veterinarians are the top candidates to deal with the inappropriate attitudes of farmers. Most farmers had good attitudes towards antibiotic storage and examination before use. However, almost 20% believed that in an emerging situation, an expired antibiotic can be used if its appearances are still normal. This misconception can lead to an underdosing treatment, which consequently causes antimicrobial resistance [24]. 70% of dairy farmers were concerned with antimicrobial resistance in a US study [13], while 94% in the present study were concerned with this situation if they were advised by a veterinarian. This finding implies that farmers would cooperate well with the intervention to impede antimicrobial resistance.

### 4.4. Practice

Although the mean total practice score of 78% seems to be sufficient, improving antibiotic use by farmers is requisite. When sick cattle were detected, the request for advice from a veterinarian was the first priority of 30% of our respondents, which is very close to the proportion reported in a US study [25]. Encouraging farmers to consult a veterinarian before treatment is important to ensure prudent antibiotic use. However, consultation without an adequate history and careful examination of yourself may not be sufficient for proper treatment [3].

Fifty-six percent of our respondents reported first treating sick animals themselves without consulting a veterinarian. This is not different from the proportion of farmers who reported self-treatment in previous studies [3, 15]. However, in Ethiopia, 97% of farmers reported that sick animals were treated by themselves [16]. The veterinarian fee is a major additional cost for veterinary treatment in addition to the drug cost, which was previously reported as a barrier to requesting veterinary help [13, 15, 16, 26, 27]. The convenient availability of drugs was reported to be another reason farmers did not need help from veterinarians [19, 26, 27]. The very high proportion of farmers who purchased antibiotics themselves (97%) indicates that this issue had also been a considerable problem in the study areas. Therefore, the endorsement of a strict regulation to control the accessibility of antibiotics may be necessary. The use of antibiotics prescribed by veterinarians may be another approach to reduce irrational practice by farmers [26–28]. Waiting for veterinary assistance can be harmful to animals in emerging situations. Thus, immediate treatment has to be administered by farmers [13, 19]. These three rationales might support the widespread self-treatment of farmers found in the present study. The inaccessibility of a veterinarian in the area was a reason for this inappropriate practice [4, 26, 27]. However, this is less likely to be cited by our respondents as an underlying cause because all participating farms were supervised by a KUVTH. Previous studies suggested that the use of irrational antibiotics by farmers can be minimized by providing comprehensive and specific protocols to treat sick animals [3, 23].

As healthcare professionals are reliable providers of antibiotic knowledge on human health [18], farmers considered veterinarians reliable providers of information for antibiotic use [14, 17, 20]. Although consultation was not regularly conducted on sick cattle treatment, most of our respondents (64%) relied on the suggestion of veterinarians for the purchase decision of antibiotics. The prescribing practices of physicians depended on their knowledge and attitudes [11], which was similar in the veterinary circumstance [12]. Continuing education was useful for improving the knowledge of physicians about antibiotics [29]. Therefore, veterinarians should regularly refresh their knowledge to ensure they can provide appropriate antibiotic advice [14, 26]. When purchasing an antibiotic, 26% of our respondents made a decision based on their experience, which was greater than the 10% reported in a previous study [3]. A minority of our respondents (7%) consulted other farmers before buying an antibiotic, which is comparable to a previous survey [26]. Drug sellers were reported as one of the important antibiotic information providers in Bangladesh [4]; however, only 3% of our respondents relied on sellers when purchasing antibiotics. In Thailand, most of the retail livestock drug stores were not under veterinarians' responsibility; therefore, they could not be trustworthy sources. The provision of adequate information on antibiotic prescribing to drug sellers would be helpful for rational antibiotic use by farmers.

The antibiotics most commonly used on dairy farms varied by area. However, penicillin class antibiotics were reported as common antibiotics used in all studies [2–4, 13, 16, 27], including in the present study. This might be due to the fact that this class of antibiotics is effective for a variety of common bacterial diseases in cattle. The use of broad-spectrum antibiotics should be avoided to delay the development of antimicrobial resistance. In Sweden, 50% of conventional dairy farmers reported using narrow-spectrum antibiotics for farm treatment, while 19% used broad spectrum [14]. However, all of the most common antibiotics used by our respondents are broad spectrum. Broad-spectrum antibiotics were ordinarily used in dairy farms by Peruvian veterinarians [3]. All common antibiotics used by our respondents had been widely available in retail drug stores in the study areas. Therefore, high availability could be a reason to select antibiotics as previously reported [3, 26]. In addition, these antibiotics have been frequently used by KUVTH veterinarians. Antibiotics with short withdrawal times and high efficacy were more likely to be used in Tennessee, USA [13]. In small-holder farms, the price of antibiotics was also considered for selection [3, 6].

In dairy farms, farmers usually try to use the least antibiotics as possible to minimize extra budget, amount of milk discarded, and time spent preventing the entry of antibiotics into the human food chain [22]. Approximately 47% of the cattle on the participating farms were treated with antibiotics each year. This proportion was similar to a survey in Peru [3]. However, these self-report data may be inaccurate. Collecting the used package is an alternative approach to quantify a more accurate amount of antibiotic use, especially for long-term data collection [3]. Antibiotics are helpful only for illnesses associated with bacterial infection. Unfortunately, 25% of our respondents reported using antibiotics on all cattle they found sick. This links to the fact found in the knowledge section that some farmers did not exactly know what antibiotics are used for. However, it is better compared to the 71% of farmers who believe that an antibiotic should be administered to all sick animals in a previous survey [19]. The mean proportion of sick cattle treated with antibiotics in the present study is obviously lower than that of a previous study (53% vs. 84%) [3]. Antibiotics should not be kept in high temperature (>30°C) and high relative humidity (>60%) conditions to ensure stability [30]. Thailand has a tropical climate, which may sometimes not achieve a suitable temperature and humidity for antibiotic storage without an additional facility, such as an air conditioner or refrigerator. The proportion of farmers who stored antibiotics in a barn in the present study (44%) was close to the 41% found in a Turkish study [15]. It is obviously greater than a previous study in Bangladesh (11%) [4], while 67% of Malaysian farmers kept antibiotics in a barn [19]. Not many farmers stored antibiotics in their homes, both in the present study (10%) and in a previous study (7%) [15]. Less than a third of farmers in Eastern Turkey reported storing antibiotics in a refrigerator [15], while it was 76% in the present study. Almost all of our respondents (96%) kept their antibiotics away from sunlight, which is higher than the 80% reported in a previous study [19]. Antibiotic storage practices complied with knowledge on this aspect. The appearance of some stored antibiotic packages indicates that they might be exposed to high humidity and physical contamination. This is consistent with our finding in the knowledge section that most respondents did not recognize the effects of humidity and physical contamination on antibiotic stability. Our respondents stored antibiotics for a shorter period compared to a previous study in which 39% (vs. 58%) and 34% (vs. 10%) of farmers stored antibiotics for 1 month and more than 4 months, respectively [15]. Most of our respondents demonstrated good practices in determining antibiotic stability prior to use by examining the expiration date or appearance. However, in relation to the misconception about the use of deteriorated antibiotics found in the attitude section, some farmers used deteriorated antibiotics, especially in emergency situations.

Local veterinarians have experienced difficulty dealing with some cases that had previously been treated by farmers. It might result from resistances of microorganisms to antibiotics. Furthermore, based on laboratory results from the Kasetsart Veterinary Diagnostic Center, antimicrobial resistances were widespread in bacteria isolated from raw milk samples submitted by farmers in the study area, including some of the respondents. The irrational use of antibiotics by farmers may partly be responsible for these antimicrobial resistances. Drug-resistant microorganisms can be transmitted to humans and other animals. Therefore, proper use by farmers should be encouraged to ensure the sustainable efficacy of antibiotics for human and animal benefit.

### 4.5. Factors Associated with Knowledge, Attitude, and Practice

The association between education and total antibiotic knowledge score found in the present study was consistent with several studies [3, 15, 16, 18]. Encouraging people with a high level of education to participate in livestock farming may be an approach to improve antibiotic knowledge in this sector [15]. The farmer who had experienced antibiotic training received a higher total knowledge score compared to farmers who had never participated in training. Based on the coefficients in the final model, the impact of this factor was greater compared to other factors. This finding indicated the effectiveness of training, which could be used to promote antibiotic knowledge and also to raise awareness of the prudent use of antibiotics in farmers. Observing farmers' practices after participating in the courses and monitoring antimicrobial resistance profiles would be helpful to determine the achievement of knowledge provision.

The influence of the hospital supervising the farm on the total knowledge score of the farmers may be responsible for the dairy cooperatives to which they were belonging. The farms under KUVTH Kamphaeng Saen and KUVTH Nong Pho supervision were mostly members of different dairy cooperatives. The dairy cooperative to which most farmers under KUVTH Kamphaeng Saen belonged to had not been vigorous and might not have devoted much attention to knowledge provision and strict regulation on antibiotic residues. Therefore, better knowledge was found in farmers supervised by KUVTH Nong Pho. Strengthening impotent dairy cooperatives and milk collection centers might be a useful intervention to secure antibiotic stewardship of farmers.

In the present study, the length of dairy farming experience was negatively associated with the total attitude score. This finding is comparable to a previous study reporting that farmers who have a longer experience than ten years less recognized the importance of strategies related to reducing antimicrobial resistance compared to farmers with shorter experience [19]. This could be due to the fact that new farmers were more likely to accept learning [26], which could improve their attitudes. Periodic refreshing courses will be necessary to arouse inactive long-experience farmers. Significant associations between attitude, education level, and age were previously reported [16]. In the present study, based on univariate analysis, these associations were not significant (*p*=0.706 and 0.135, respectively).

The advice of nonveterinarians could lead to improper use of antibiotics [31]. Not surprisingly, in the present study, the association was found between the knowledge of other dairy farmers and the low total practice score. Accessible advisement from veterinarians might restrain farmers from consulting nonveterinarians. Although the impacts of education, age, and gender on the antibiotic use behaviors of farmers were previously recognized [16], in the present study, these univariate associations were far from significant (*p*=0.913, 0.480, and 0.705, respectively).

Knowledge, attitude, and practice on antibiotic use have been considered important elements of prudent antibiotic use [16]. The adjustment of knowledge and attitude is plausible; therefore, changing them is considered an attractive strategy to improve antibiotic use [11, 14]. This suggestion is in accordance with the link between knowledge, attitude, and practice in our respondents. The positive associations between knowledge and attitude found in the present study were consistent with a previous report [16]. A significant association was observed between knowledge and practice on antimicrobial use and resistance in several studies [15, 16, 23]. Although it did not exist in the present study, improving farmer knowledge should not be ignored because knowledge could indirectly influence practices through attitudes. Attitude has been considered as a major determinant of achievement [16, 23]. In the present study, a significant association was detected between attitude and practice. This association was also reported by a study on antibiotic use conducted in Thai pharmacists [32]. The finding indicated an important modification of attitudes towards the rational use of antibiotics. Veterinarians have been expected to change farmers' attitudes [23]. Therefore, a good farmer-veterinarian relationship is necessary for the proper use of antibiotics by farmers.

### 4.6. Possible Further Studies

It would also be of interest to monitor knowledge, attitudes, and practices of farmers after implementation of our suggested interventions. Although evidence of antibiotic misuse by farmers has been available, the proof that their misuses aggravate antimicrobial resistance remains a deficit. Information on actual antibiotic use practices by farmers and the antimicrobial resistance situation in the study area should be closely examined. The link between these kinds of information would be favorable to affirm that farmers are stimulants of antimicrobial resistance. Moreover, significant erroneous practices will be identified for legitimate correction.

### 4.7. Limitations

The most likely bias in the present study is the social desirability bias. However, the objective of the study was clearly explained to respondents, and truthful responses were requested to minimize this bias. In addition, a clear instruction was provided to interviewers with the questionnaire. Some questions in the present study, especially those that asked for a number in the practice section, might be affected by recall bias. However, interviewers could help overcome this obstacle by adapting the questions to an easier way to recall the answer. Because some respondents did not know exactly what antibiotic is, there might be a misunderstanding in some questions. Consequently, a deviation from reality might exist.

No concrete criterion has been available to justify attitudes and practices as good or bad. Thus, the justifying was relied on by researchers. This study was conducted in farms under the supervision of KUVTH, for which a regular veterinarian visit was scheduled. Farmers might have a closer relationship with veterinarians compared to other farmers, which could influence their knowledge, attitudes, and practices. Therefore, the generalizability of the study may be limited. Additionally, due to the nature of the cross-sectional study, the association found in the present study cannot imply causation.

## 5. Conclusions

Farmers used antibiotics extensively on participating farms. The level of knowledge about antibiotics from farmers was moderate, while their attitudes and practices were acceptable. Therefore, knowledge on antibiotic and antimicrobial resistance needs to be enhanced to improve attitudes and practices. Training seems to be a powerful way to pass on knowledge to farmers. Because veterinarian was cited as a trustworthy information provider, strengthening the farmer-veterinarian relationship is probably useful to promote prudent antibiotic use of farmers. Encouraging young people with higher education to participate in dairy farming may be an alternative approach to improve antibiotic use in the industry. Providing antibiotic use protocols and decreasing the availability of antibiotics should also be considered to limit overuse by farmers.

## Figures and Tables

**Table 1:** Frequency distribution of data in the general information of farm and farmer section from 89 dairy farmers.

Question	Category	Percentage of participants
Gender of respondents	Male	65.17
	Female	34.83

Education of respondents	Uneducated	2.25
	Grade 4	7.87
	Grade 6	22.47
	Grade 9	8.99
	Grade 12	30.34
	Bachelor degree	25.84
	Master degree	2.25

Source of knowledge on antibiotic use	Veterinarians of Kasetsart University Veterinary Teaching Hospital	93.26
	Other dairy farmers	57.30
	Retailer of the drug store	56.18
	Training course	41.57
	Printed materials	37.08
	Staffs of the Department of Livestock Development	33.71
	The Internet	26.97
	Staffs of dairy cooperation	7.86
	Private veterinarians	5.62
	Drug labels	4.49
	Other institutions	3.37
	Artificial inseminators	3.37
	Media	2.25

**Table 2:** The final multivariate regression model identifying factors associated with the total knowledge score obtained from 88 dairy farmers^*∗*^.

Variable	Coefficient	Standard error	*p* value	95% confidence interval	
Education					
Grade 6 or lower					
Grade 9 to 12	3.044	1.391	0.031	0.277	5.811
Bachelor degree or higher	3.812	1.494	0.013	0.840	6.784
Participating in a training related to antibiotics					
No					
Yes	4.046	1.208	0.001	1.642	6.450
Supervising hospital					
Kamphaeng Saen					
Nong Pho	3.502	1.426	0.016	0.665	6.340
					
Total attitude score	1.611	0.683	0.021	0.252	2.970

^*∗*^One farmer did not complete all required questions in the knowledge section.

**Table 3:** The final multivariate regression model identifying factors associated with the total attitude score obtained from 76 dairy farmers^*∗*^.

Variable	Coefficient	Standard error	*p* value	95% confidence interval	
Year of experience on dairy farming					
<20					
≥20	−0.417	0.176	0.020	−0.767	−0.066
					
Total practice score	0.328	0.072	<0.001	0.183	0.473

^*∗*^Thirteen farmers did not complete all required questions in the attitude section.

**Table 4:** The final multivariate regression model identifying factors associated with the total practice score obtained from 76 dairy farmers^*∗*^.

Variable	Coefficient	Standard error	*p* value	95% confidence interval	
Obtaining knowledge on antibiotics from other dairy farmers					
No					
Yes	−0.704	0.243	0.005	−1.189	−0.220
					
Total attitude score	0.654	0.135	<0.001	0.381	0.927

^*∗*^Thirteen farmers did not complete all required questions in the practice section.

## Data Availability

The data used to support the findings of this study are available from the corresponding author upon request.
